# Self‐care behavior prevention of COVID‐19 in the general population based on Pender Health Promotion Model: A cross‐sectional study

**DOI:** 10.1002/hsr2.894

**Published:** 2022-10-17

**Authors:** Asghar Pouresmali, Shahriar Dargahi, Jaber Alizadehgoradel, Aziz Kamran, Davod Fathi, Behnam Molaei

**Affiliations:** ^1^ Department of Family Health, Social Determinants of Health Research Center Ardabil University of Medical Sciences Ardabil Iran; ^2^ Department of Counseling University of Mohaghegh Ardabili Ardabil Iran; ^3^ Department of Psychology, Faculty of Humanities University of Zanjan Zanjan Iran; ^4^ Department of Health Education and Promotion, School of Medicine Ardabil University of Medical Sciences Ardabil Iran; ^5^ Department of Psychiatry, Faculty of Medicine Ardabil University of Medical Sciences Ardabil Iran

**Keywords:** commitment to action, COVID‐19, Pender's Health Promotion Model, self‐care

## Abstract

**Background and Aims:**

Coronavirus with its sudden and widespread outbreak has obviously imposed devastating consequences in various aspects of human life. The purpose of this study was to determine the predictive value of Pender's Health Promotion Model (HPM) structures in self‐care preventive behavior against coronavirus disease 2019 (COVID‐19) among the general population of Ardabil, Iran.

**Methods:**

The present retrospective descriptive‐correlational study was conducted on citizens of Ardabil aged 18 years and over in 2021. After dividing the city of Ardabil into four parts, 50 people from each area of the city and a total of 200 people were selected through the available sampling method through social media. Data collection tools included a demographic profile, perceived self‐efficacy scale, perceived emotional questionnaire, perceived social support questionnaire, perceived benefits and barriers questionnaire, researcher‐made COVID‐19 self‐care questionnaire, and commitment to action questionnaire based on Pender's HPM structures in an online manner. Data were analyzed by Amos 22 software and using structural equation modeling.

**Results:**

According to the results, direct path analysis to COVID‐19 self‐care behavior indicated that the variables of perceived self‐efficacy (*β* = 0.18, *p* < 0.01), interpersonal effects (*β* = 0.19, *p* < 0.01), positive emotion (*β* = 0.15, *p* < 0.05) and perceived benefits (*β* = 0.20, *p* < 0.01) are able to significantly predict self‐care behaviors. Moreover, the bootstrapping test results in the indirect path analysis demonstrated that the variables of perceived self‐efficacy (95% confidence interval [CI], 0.012, 0.066), perceived social support (95% CI, 0.002, 0.026), and perceived barriers (95% CI, −0.019, −0.002) and benefits (95% CI, 0.001, 0. 015) through the mediator variable of commitment to action are able to significantly predict COVID‐19 self‐care behavior.

**Conclusions:**

Based on the findings of the present study, it can be claimed that the proposed model of COVID‐19 self‐care behavior has an acceptable fitness in the general population. This model can be used in developing educational programs and intervention techniques to modify people's attitudes and behaviors.

## 1 BACKGROUND

Coronavirus disease 2019 (COVID‐19) is an emerging infectious disease developed by severe acute respiratory syndrome coronavirus 2 from the coronavirus family and spreads through saliva droplets or nasal secretions during coughing or sneezing.^1^, ^2^ Following the onset of the disease in December 2019, it has spread rapidly throughout the world due to the very high rate of spreading of the causative agent of the disease and has become a global crisis in almost a short time, in less than 4 months.^3^ In January 2020, the World Health Organization (WHO) issued a statement declaring the novel coronavirus to be the sixth leading cause of public health emergency worldwide.^4^ After the announcement of the COVID‐19 pandemic, the virus control approach in all countries was seriously included in the agenda of governments and international health officials.^5^, ^6^

Adherence to self‐care behaviors and hygiene protocols (including the use of face masks and gloves, social distancing, avoidance of unnecessary travel, and hand washing with soap and water) was critical to control the COVID‐19 pandemic. Research has also confirmed the need to follow health tips and the importance of self‐care in reducing the risk of COVID‐19.^7^ In this regard, limiting the number of hospitalized people and duration of hospitalization, banning direct visits, providing physical care and psychological assistance,^8^ encouraging citizens to engage in self‐care behaviors, such as working at home, staying at home, and canceling unnecessary meetings are some of the effective prevention and treatment strategies used to control the spread of the disease.^9^

Various studies revealed that self‐care behaviors improve people's quality of life.^10^ One of the theories based on the promotion of human physical and mental status is the theory of “empowerment.”^11^ According to this theory, the most important component that can lead to empowerment is self‐care behavior.^12^

Health Promotion Model (HPM) is one of the influential factors in choosing self‐care behaviors and thus improving quality of life, which affects a person's behaviors. Pender's HPM is one of the most comprehensive and defining patterns of health promotion behaviors in the general population. This model was introduced in 1996 as a framework for identifying and modifying unhealthy behaviors and promoting health. The reason for emphasizing the use of the constructs of this model is its comprehensiveness and application in recognizing the determinants of behavior. Predicting factors and explanatory constructs of health behavior in Pender's model include perceived benefits, perceived barriers, perceived self‐efficacy, behavioral emotions, interpersonal influencers, situational influencers, and commitment to action.^13^ Self‐care must be learned and performed intentionally and permanently. Learning self‐care activities can lead a person to stay healthy and well, increase a person's adaptation to illness, increase self‐care capacity, and reduce patients' disability and treatment costs.^14^

Despite the implemented policies such as media advertising, education, legal barriers, and encouraging people to increase self‐care behaviors, according to the studies conducted, despite the high level of self‐care behaviors among special classes, it seems that health and self‐care behaviors are at a low level among the general population of Iran.^1^ Considering the widespread and rapid spread of COVID‐19 in the world, including Iran, it can be said that self‐care behaviors are one of the key factors in preventing this disease, which has a deep connection with psychosocial issues^15^ and has been less addressed from a psychosocial perspective. In this regard, the researchers in the present study, by identifying the causes and factors affecting adherence to self‐care behaviors and emphasizing the empowerment structures, the need to pay more attention to education, design, planning, and implementation of comprehensive programs to increase self‐care behaviors is felt. Therefore, the present study used Pender's HPM framework to determine and identify factors affecting preventive self‐care measures against COVID‐19 among the general population of Ardabil city in 2021.

## 2 MATERIALS AND METHODS

The current retrospective descriptive‐correlational study was conducted on the statistical population of the study, including the general population over 18 years of age in Ardabil, Iran in 2021. According to Hoelter,^16^ the sample size must be bigger than the covariate matrix, and it must be at least a 5:1 ratio for the number of subjects to the number of the model parameters, but a 10:1 ratio is recommended. If the observed variables are 12 or less, then a minimum of 200 samples are necessary. In the hypothetical model of this study, the number of observed variables is 9.

In this regard, 200 people were selected by convenience sampling method. First, the city of Ardabil was divided into four parts and 50 people from each part were selected according to the age criteria (above 18 years). Due to the limitations of the COVID‐19 epidemic, after explaining the objectives of the research and receiving contact numbers from people to send the link to the questionnaires and the answer guide, data were collected by electronic questionnaire and in cyberspace from the research units who met inclusion criteria, including living in Ardabil, being at least 18 years old, having at least eighth‐grade educational level, willingness to cooperate in the study, not being treated due to serious physical and mental illness, and also no history of cognitive disorders or specific physical limitations. In the present study, the questionnaires were completed by observing ethical considerations and informed consent was filled out to participate in the study. The confidentiality of information, the study methodology and objectives, and the method of self‐report answering the questions were explained to the participants.

### 2.1 Data collection tools

#### 2.1.1 Demographic questionnaire

The required information included gender, age, marital status, educational level, occupational status, and the number of family members.

#### 2.1.2 COVID‐19 self‐care questionnaire

*Self‐care questionnaire*: It is designed by researchers. It contains 17 questions and has been designed with score 5 as “always” and “not at all” with score of 1. The preparation of items has been designed by a qualitative interview and the use of theoretical literature. For the study of tool validity, three methods of content validity, face validity, and construct validity were used. First, the content validity of the items was investigated using Lawshe's method. For the quantitative study of content validity, both the content validity ratio (CVR) and content validity index was used. To determine the CVR, 11 health professionals and caregivers were asked to select each item based on the three‐part spectrum: “necessary,” “useful but not necessary,” and “not necessary,” and then to re‐examine each item on the basis of four options: “not relevant,” “the need for serious review,” “relevant but need to review,” “completely relevant.” After collecting the experts' opinions and analyzing the results, the CVR of all self‐care questionnaire questions was calculated. The obtained results show the coordination between the content of measurement tools and the survey's objects. Using the method of factor analysis (exploratory), the construct validity of the self‐care questionnaire was investigated. Before the exploratory factor analysis, the Kaiser–Mayer–Olkin test and Bartlett's test of sphericity were performed. The coefficient of the Kaiser–Mayer–Olkin test was 0.82, which showed that the sample size was satisfactory for the factor analysis. Also, Bartlett's test of sphericity (879/801) was meaningful at the level of 0.0001, which indicates that the factor analysis method is appropriate for identifying the structure of the factor model. To determine the number of factors in the self‐care questionnaire, graphical methods such as very simple structure, parallel analysis scree plots, specific value, and variance explained by each factor were used. The conclusion of this study suggested a two‐factor model that was consistent with the theoretical foundations of the research. To conduct the factor analysis of this questionnaire, the maximum likelihood method was used along with Promax Rotation. The results of exploratory factor analysis showed that 5, 11, 13, 14, 15, and 16 had a very low factor load on both factors that were excluded from the analysis process. After removing the mentioned items, the exploratory factor analysis was repeated. Examination of the results showed that the two factors together accounted for 53% of the total variance, with a share of 29% and 24% of the variance for the first factor and the second factor, respectively. According to Cronbach's *α* method, the estimated reliability of Factor 1 (Personal Care) and Factor 2 (Social Responsibility) were 0.71 and 0.83.

#### 2.1.3 Perceived Self‐Efficacy Scale

This questionnaire was according to Smith et al. In this questionnaire, eight questions were designed based on the 5‐point Likert scale from “strongly agree” to “strongly disagree.” A higher score indicated that the individual had a higher ability to control the results and outcomes of health‐related programs. The Cronbach's *α*‐coefficient reported by Smith et al.,^17^ for this instrument, was 0.84. In Iran, in the study of Bayat et al.,^18^ Cronbach's *α*‐coefficient of 0.73 has been obtained. The coefficient of the sample adequacy index (KMO) is also above 0.80, which shows that the instrument has a suitable validity.^18^ In the present study, Cronbach's *α*‐coefficient to evaluate the reliability of the test was estimated at 0.87.

#### 2.1.4 Perceived Emotions Questionnaire

This questionnaire was adapted from the tools of Watson et al.^19^ There are 20 questions scored on the 5‐point Likert scale from “almost never” to “almost always.” This tool measures the two subscales of positive emotion and negative emotion and each subscale have 10 items. Cronbach's *α*‐coefficient reported by Watson et al. was 0.94 for positive emotions and 0.91 for negative emotions. In this study, Cronbach's *α*‐coefficient for positive and negative emotions was 0.79 and 0.83, respectively. In Iran, in the study of Bayat et al.,^18^ Cronbach's *α*‐coefficient of 0.71 has been obtained. The coefficient of the sample adequacy index (KMO) is also above 0.80, which shows that the instrument has a suitable validity.

#### 2.1.5 Perceived Social Support Questionnaire

This questionnaire was adapted from Kanti et al.^20^ In this questionnaire, 12 questions were designed based on the 7‐point Likert scale from “strongly agree” to “strongly disagree”. Higher scores indicate more support from friends, family, and other important people. The Cronbach's *α*‐coefficient reported by Kant et al. was 0.91 for this instrument. In this study, Cronbach's *α*‐coefficient was estimated at 0.90 to evaluate the reliability of the test. In Iran, in the study of Bayat et al.,^18^ Cronbach's *α*‐coefficient of 0.76 has been obtained. The coefficient of the sample adequacy index (KMO) is also above 0.80, which shows that the instrument has a suitable validity.

#### 2.1.6 Perceived Barriers Questionnaire

This scale was adapted from Becker et al.^21^ In this questionnaire, 18 questions were designed based on the 4‐point Likert scale from “never” to “always.” A higher score indicated that the respondent faced more barriers to performing health‐promoting behaviors. Cronbach's *α*‐coefficient reported by Becker et al. was 0.80 and the test–retest coefficient was 0.75 for this instrument. Cronbach's *α*‐coefficient for test reliability was 0.76. In Iran, in the study of Bayat et al.,^18^ Cronbach's *α*‐coefficient of 0.73 has been obtained. The coefficient of the sample adequacy index (KMO) is also above 0.80, which shows that the instrument has a suitable validity.

#### 2.1.7 Perceived benefits

The perceived benefits construct was evaluated using an assessment tool adapted by Mohammadian et al.,^22^ which consisted of 20 items scored based on a 4‐point Likert scale. This tool assesses the anticipated positive outcomes that will occur from health behavior. The score obtained ranges from 20 to 80 and higher scores indicate more benefits perceived for health‐promoting behaviors. Internal consistency reported by Mohammadian et al.^22^ for this instrument was good. Internal consistency for the current study was 0.79.

#### 2.1.8 Situational influences

To evaluate the situational influences, we designed a questionnaire based on Pender's model,^13^ which included seven items. This instrument assesses the personal perceptions and cognitions of any given situation or context that can facilitate or impede behavior. The items were rated on a four‐choice Likert scale ranging from “never (1)” to “always (4).” The total scores ranged from 7 to 28. The higher score indicates a higher level of situational influences. In the current research, Cronbach's *α*‐coefficient for this tool was 0.78. In Iran, in the study of Bayat et al.,^18^ Cronbach's *α*‐coefficient of 0.75 has been obtained. The coefficient of the sample adequacy index (KMO) is also above 0.80, which shows that the instrument has a suitable validity.

#### 2.1.9 Commitment to action

To assess the “commitment to action,” we developed a tool, based on Pender's theory, that evaluates the intention and identification of a planned strategy that leads to the implementation of health behavior.^13^ This assessment tool contains 12 items and is scored based on a 5‐point Likert scale, ranging from strongly agree (5) to strongly disagree (1). The score obtained for each item range from 10 to 50. Higher scores indicate better‐reported commitment to action. In Iran, in the study of Bayat et al.,^18^ Cronbach's *α*‐coefficient of 0.72 has been obtained. The coefficient of the sample adequacy index (KMO) is also above 0.80, which shows that the instrument has a suitable validity. In the present research, Cronbach's *α*‐coefficient for this tool was 0.86.

### 2.2 Data analysis

Data were analyzed using Amos 22 software and direct and indirect path analysis with structural equation modeling.

## 3 RESULT

Table 1 shows the demographic information of the participants, including age, sex, marital status, employment, education, and the number of family members of the participants.

**Table 1:** Sociodemographic characteristics of the sample (*n* = 200)

Variable	*N*	%
Sex		
Female	80	40
Male	120	60
Age (years)		
18–25	72	36
26–33	68	34
34–41	38	19
42–49	14	7
50 or over	8	4
Marital status		
Married	102	51
Single/divorced/widowed	95	47.5
Did not answer	3	1.5
Education		
Up to grade 12	35	17.5
Bachelor's degree	86	43
Master's/Doctoral degree	75	37.5
Did not answer	4	2
Employment		
Nongovernmental	72	36
Governmental	69	34.5
Student	5	2.5
Homemaker	20	10
Retired/unemployed	34	17
Family members		
1–3	78	39
4–6	107	53.5
>6	15	7.5

### 3.1 Correlational analysis

Table 2 shows commitment to action (*r* = 0.34, *p* < 0.01), perceived self‐efficacy (*r* = 0.28, *p* < 0.01), interpersonal in influences (*r* = 0.29, *p* < 0.01), perceived social support (*r* = 0.21, *p* < 0.01), positive affect (*r* = 0.30, *p* < 0.01), and perceived benefits (*r* = 0.32, *p* < 0.01) have positive relationship with COVID‐19 self‐care behaviors. Therefore, with an elevation of commitment to action, perceived self‐efficacy, interpersonal influences, perceived social support, positive affect, and perceived benefits, COVID‐19 self‐care behaviors increases.

**Table 2:** Mean (*M*), standard deviation (SD), and Pearson's correlation coefficients among the variable (*n* = 200)

Variables	1	2	3	4	5	6	7	8	9
COVID‐19 self‐care behaviors	1								
Commitment to action	0.34**	1							
Perceived self‐efficacy	0.28**	0.24**	1						
Interpersonal influences	0.29**	0.17*	0.13	1					
Perceived social support	0.21**	0.25**	0.00	0.29**	1				
Positive affect	0.30**	0.11	0.10	0.32**	0.23**	1			
Negative affect	0.02	−0.02	0.10	−0.19**	−0.15*	−0.05	1		
Perceived barriers	−0.12	−0.17*	0.15*	−0.33**	−0.24**	−0.22**	0.38**	1	
Perceived benefits	0.32**	0.22**	0.02	0.37**	0.36**	0.40**	−0.37**	−0.32**	1
Mean	61.10	53.16	29.76	21.91	52.70	34.31	20.50	30.10	50.33
SD	4.14	4.17	3.03	3.54	6.71	8.85	5.70	9.00	10.29

Correlation is significant at the *0.01 and **0.05 levels (two‐tailed).

### 3.2 Model fitness

Table 3 shows fit indices. According to the results, the modified model had sufficient goodness‐of‐fit: CMIN/DF (normed *χ* ^2^) = 1.33, goodness‐of‐fit index = 0.99, adjusted goodness‐of‐fit index = 0.92, incremental fit index = 0.99, Tucker–Lewis index = 0.96; comparative fit index = 0.99, root mean square error of approximation = 0.04.

**Table 3:** Fit indices of the modified model

Modification indexes	CMIN/DF	GFI	AGFI	IFI	TLI	CFI	RMSEA
Suggested value	1–5	>0.90	>0.80	>0.90	>0.90	>0.90	<0.08
Modified model	1.33	0.99	0.92	0.99	0.96	0.99	0.041

Abbreviations: AGFI, adjusted goodness‐of‐fit index; CFI, comparative fit index; CMIN/DF, normed *χ* ^2^; GFI, goodness‐of‐fit index; IFI, incremental fit index; RMSEA, root mean square error of approximation; TLI, Tucker–Lewis index.

**Figure 1:**
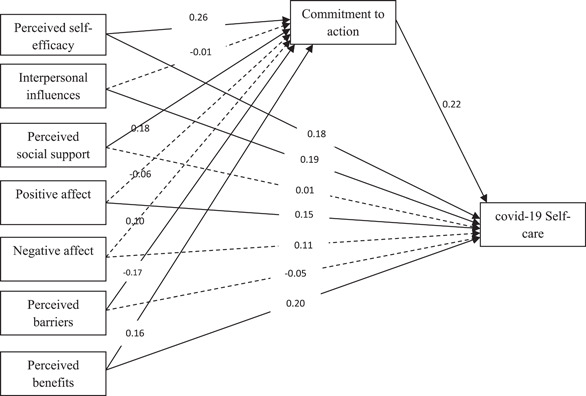
Standard regression coefficients (*β*'s) of mediation of commitment to action between predictive variables and self‐care coronavirus disease 2019 (COVID‐19)

According to Figure 1, the results show that perceived benefits and perceived self‐efficacy variables, in addition to their direct effect and mediating effect, are also confirmed. That is, with the inclusion of the mediator variable, their direct relationship with the dependent variable remains significant (partial mediation). Also, perceived barriers and perceived social support variables have an effect on the dependent variable only through the mediator variable (full mediation).

### 3.3 Paths coefficients

The standard coefficients of direct pathway in Figure 1 indicate that direct perceived self‐efficacy pathway to COVID‐19 self‐care (*β* = 0.18, *p* < 0.01), interpersonal in influences pathway to COVID‐19 self‐care (*β* = 0.19, *p* < 0.01), positive affect pathway to COVID‐19 self‐care (*β* = 0.15, *p* < 0.05), perceived benefits pathway to COVID‐19 self‐care (*β* = 0.20, *p* < 0.01), perceived self‐efficacy pathway to commitment to action (*β* = 0.26, *p* < 0.01), perceived social support pathway to commitment to action (*β* = 0.18, *p* < 0.01), perceived barriers pathway to commitment to action (*β* = −0.17, *p* < 0.05), pathway of perceived benefits to commitment to action (*β* = 0.16, *p* < 0.05), and the pathway of commitment to action to COVID‐19 self‐care (*β* = 0.22, *p* < 0.01) were significant.

### 3.4 Indirect effects

According to Table 4, the results of bootstrapping analysis showed that commitment to action had a partially mediated relationship between perceived self‐efficacy and COVID‐19 self‐care (95% confidence interval [CI], 0.012, 0.066), since zero is outside the confidence interval of 0.95, so this path is significant. The indirect path from perceived social support to COVID‐19 self‐care through commitment to action was significant (95% CI, 0.002, 0.026). Commitment to action fully mediated this relationship. Commitment to action had a full mediation effect on the relationship between perceived barriers and COVID‐19 self‐care (95% CI, −0.019, −0.002). The indirect path forms perceived benefits to COVID‐19 self‐care through commitment to action was partially significant (95% CI, 0.001, 0.015).

**Table 4:** Bootstrap results for indirect effects

Independent variable	Mediator	Dependent variable	Number of bootstrap samples	Lower	Upper	Confidence interval
Perceived self‐efficacy	Commitment to action	COVID‐19 self‐care	1000	**0.012**	**0.066**	0.95
Interpersonal influences	Commitment to action	COVID‐19 self‐care	1000	−0.016	0.019	0.95
Perceived social support	Commitment to action	COVID‐19 self‐care	1000	**0.002**	**0.026**	0.95
Positive affect	Commitment to action	COVID‐19 self‐care	1000	−0.012	0.002	0.95
Negative affect	Commitment to action	COVID‐19 self‐care	1000	−0.001	0.020	0.95
Perceived barriers	Commitment to action	COVID‐19 self‐care	1000	**−0.019**	**−0.002**	0.95
Perceived benefits	Commitment to action	COVID‐19 self‐care	1000	**0.001**	**0.015**	0.95

*Note*: Bold numbers are paths that are significant at the 0.05 level.

In addition, the results showed that the indirect effect of interpersonal influences and positive affect and negative affect on COVID‐19 self‐care via commitment to action is not significant, since zero is not outside the confidence interval of 0.95. To study the indirect effects, the Bootstrap test was used, and its results are presented in Table 4.

## 4 DISCUSSION

The present study aimed to determine the predictive value of Pender's HPM structures in performing COVID‐19 self‐care behaviors in the general population.

Our results revealed that the perceived self‐efficacy was both directly and indirectly able to predict COVID‐19 self‐care behavior through commitment to action. It should be noted that 86% of studies on health‐promoting model supported the importance of self‐efficacy as a determinant of health‐promoting behavior.^15^, ^23^ Self‐efficacy has been emphasized as a significant precondition for self‐management to improve health‐promoting behaviors.^24^, ^25^ Other studies found that perceived self‐efficacy influences a person's ability to perform a particular level of action, and commitment to action is affected by variables such as perceived self‐efficacy.^13^

Based on the results, interpersonal communication is directly able to significantly explain self‐care against COVID‐19, which is in line with Sun and Jiang^26^ study. In other words, people with effective interpersonal communication skills have a high level of self‐care during the COVID‐19 outbreak. The ability to communicate effectively is one of the 10 skills emphasized by the WHO and a prerequisite for mental health in individual and social life. This skill is important in human life as some experts have stated that the communication process is the basis of all human development, personal injury, and human progress.^27^ Hence, it can be said that such a skill can lead a person to take care of him/herself and others while developing mental health.

The results also showed that perceived social support could not directly predict COVID‐19 self‐care behavior, but significantly and indirectly predicted COVID‐19 self‐care behavior through commitment to action. According to the results of several studies on the effect of social and family support in reducing stress levels and improving skills in controlling the prevalence of diseases such as influenza,^28^ Ebola,^29^ SARS,^30^ and COVID‐19,^31^, ^32^ our findings also emphasized the role of social support and commitment to action in the individual as components promoting COVID‐19 self‐care behaviors. In other words, a central function of commitment to action in COVID‐19 self‐care behavior is an acceptable approach to health promotion in which people in the community are empowered to take responsibility for their own health and the health of others and to adopt a healthy lifestyle.

Based on the results, positive emotion is directly able to significantly predict COVID‐19 self‐care behavior. Positive emotion has been shown to be a key component of emotion control skills, and plays an important role in people's adaptation to stressful life events.^33^ People who have experienced negative life events but focus on the positive aspects of life report greater life satisfaction.^34^ Therefore, positive emotions and high mood can enhance people's hope and quality of life. In such cases, positive emotions will lead to the adaptation of effective control strategies and self‐care behaviors of COVID‐19. The results on the role of negative emotion in COVID‐19 self‐care are not significant because this component is unable to directly or indirectly predict COVID‐19 self‐care behavior.

Another finding of the present study is the absence of directly predicting self‐care behaviors by the variable of perceived barriers. On the other hand, the results of this study also revealed that commitment to action is indirectly able to predict COVID‐19 self‐care behavior. Most studies testing the health promotion model have expressed empirical support for the importance of barriers as a determinant of health‐promoting behavior.^35^ In such cases, perceived barriers can be imaginatively related to the inaccessibility, inappropriateness, costliness, dissatisfaction, difficulty, or time‐consuming nature of a particular action, which acts as a barrier to behavior.^13^

Contrary to previous findings, this study found no inverse correlation between perceived barriers and self‐care behaviors. In other words, barriers to self‐care practices and behaviors (such as lack of financial resources, apathy, and shortage of time) do not prevent self‐care behaviors. Such a result is not unexpected given the serious and deadly nature of COVID‐19, and it seems that when it comes to one's life, people endure deficiencies and barriers to save their lives. On the other hand, the mediating role of commitment to action in the relationship between perceived barriers and self‐care behaviors shows that although barriers alone cannot affect individuals' self‐care and preventive behaviors at COVID‐19 risk, they can influence self‐care behaviors of people by reducing their motivation and commitment. In other words, barriers have no effect on the onset of COVID‐19 self‐care behaviors, but can reduce individuals' motivation to pursue self‐care behaviors by affecting their commitment.

Based on the results of the present study, the perceived benefits of individuals can directly explain self‐care behavior and are indirectly able to predict COVID‐19 self‐care behavior through commitment to action. This result is consistent with other findings in this field.^36^, ^37^

People's perception of the positive outcomes and benefits of self‐care can increase people's motivation to increase such behaviors.^13^ Studies show that if perceived barriers outweigh the benefits of prediction, behavior is less likely to occur. In other words, the individual's action directs the self‐care behavior through the balance and imbalance between perceived positive and negative forces.^32^

The limitations of the present study are the self‐report nature and Internet completion of the designed questionnaire, which reduce the reliability of the data. To reduce these problems, an attempt was made to consider an option when designing an online questionnaire so that a user could complete the online form only once with an ID. In addition, the online form was tried to be sent through various communication channels to provide the ability to respond with smartphones as well as the operating system. However, one of the major problems with online questionnaires is the need for the Internet to complete the relevant form, which eliminates the chances of people without these facilities participating in such studies. Also, the lack of control of some variables, such as the history of being infected with COVID‐19 in oneself and the family, and occupational and social status, were among the limitations of this study.

## 5 CONCLUSION

According to the findings of the current research, it can be said that the self‐care behavior prevention of COVID‐19 based on Pender's HPM has acceptable fitness in the general population. It seems that this model can be employed in developing educational programs and intervention techniques to modify people's attitudes and behavior. It is suggested to examine other models and theories of education and behavior change such as the theory of planned behavior and the protection motivation theory regarding the adaptation of COVID‐19 self‐care behaviors.

## AUTHOR CONTRIBUTIONS

**Asghar Pouresmali**: Methodology; project administration. Shahriar Dargahi: Writing–review and editing. **Jaber Alizadehgoradel**: Resources; software; validation. **Aziz Kamran**: Project administration. **Davod Fathi**: Data curation; validation. **Behnam Molaei**: Conceptualization.

## CONFLICT OF INTEREST

The authors declare no conflict of interest.

## ETHICS STATEMENT

The present research is derived from a research project with the financial support of Ardabil University of Medical Sciences and has code of ethics number IR.ARUMS.REC.2020.040.

## TRANSPARENCY STATEMENT

The lead author Behnam Molaei affirms that this manuscript is an honest, accurate, and transparent account of the study being reported; that no important aspects of the study have been omitted; and that any discrepancies from the study as planned (and, if relevant, registered) have been explained.

## Data Availability

The data that support the findings of this study are available from the corresponding author upon reasonable request.
